# Ischemic Colitis

**Published:** 2012

**Authors:** L Dimitriu, D Ferechide

**Affiliations:** * “Carol Davila” University of Medicine and Pharmacy; Department of Internal Medicine, Colentina Clinical Hospital, Bucharest, Romania; * “Carol Davila” University of Medicine and Pharmacy, Bucharest

**Keywords:** ischemic colitis, Tollefson classification, bloody diarrhea, pancolitis

## Abstract

Ischemic colitis occurs as a result of the decrease of arterial blood supply at the level of the colon and may have consequences which vary from completely reversible lesions to ischemic necrosis. It is a condition that affects both sexes, predominantly people over the age of 50, having multiple etiologies in which the main role is played by atherosclerosis. The symptomatology varies from mild symptoms, which are reversible, to severe ones, with a vital risk. The colonoscopy does not show pathognomonic signs, but it has high sensitivity due to the involvement of the mucosa. There are different treatments according to the clinical form. In general, it goes into remission spontaneously. Emergency surgical treatment is required in acute pancolitis, toxic megacolon, massive hemorrhages, when a subtotal colectomy is performed.

It represents an anatomical and pathologic spectrum, which depends on the onset, duration, cause and localization of the segment involved, on its length and on the existence of collateral circulation [**15**].

The decrease of the arterial blood supply at the level of the colon may have consequences which vary from completely reversible lesions, noticeable only microscopically, to total ischemic necrosis of the colic wall [**25**].

The incidence of the condition is variable: 7.5/100,000 inh./year, but actually it is difficult to estimate the real incidence due to the multiple mild, reversible forms [**26**]. It is difficult to make a diagnosis because it is sometimes hard to distinguish it from colitis due to an inflammatory cause [**4**]. The condition affects both sexes and no differences based on race or ethnicity have been noted [**5**].

**Etiology**: non-iatrogenic causes:

1. diseases due to low blood flow: global heart failure, shock due to various causes, hypovolemias, rhythm disorders [**3**];

2. atherosclerosis which causes embolism or thrombosis in the superior and inferior mesenteric artery [**1,2**];

3. hematologic diseases – coagulopathies caused by AT III deficiency, Protein C, S, deficiency, Factor V Leiden, antiphospholipid syndrome, polycythemia vera [**7**];

4. vasculitis in Takayasu’s disease, giant cell arteritis, periarteritis nodosa, Churg–Strauss syndrome, thrombotic thrombocytopenic purpura [**11**];

5. vasculopathies in diabetes and amyloidosis;

6. parasitic or viral bacterial infections;

7. acute pancreatitis;

8. pheochromocytoma;

9. obstructive colonic lesions which produce secondary distension in colorectal cancer, strangulated hernia, diverticulitis [**21**].

Iatrogenic causes:

1. Medical causes: following a treatment with flutamide, immunosuppressants, AINS, saline laxatives, gold salts, diuretic medications, digitalis, penicillin, following a colonoscopy and an aortography [**9**].

2. Surgical causes: insertion of various vascular prostheses, liver transplantation, colonic by-pass [**6**].

**Tollefson Classification:** according to the severity of the disease and to the anatomical and pathologic lesions, three stages may be described:

• Stage I – in which there are edema, hyperemia and hemorrhages localized at the level of the mucosal and submucosal region, with or without ulcerations. The lesions are limited, with a favorable evolution towards healing.
• Stage II - the muscularis is involved, with necrosis at this level and the formation of fibrous tissue, leading to strictures. The lessions have a mean severity [**17**].
• Stage III – necrosis develops on the entire colonic wall, which leads to severe metabolic disturbances, with a severe prognosis. Mortality is high and can reach 60-70%.

**Clinical Diagnosis** – the symptomatology varies from mild symptoms, which are reversible, to severe ones, with a vital risk. The patients have difficult bowel movement with bloody diarrhea and abdominal pain of high and moderate intensity [**16**]. In 10% of the cases, the patients may show the signs of acute peritonitis secondary to colonic necrosis.

The physical examination reveals abdominal distension, meteorism, tenderness at palpation, a palpable abdominal mass in the left iliac fossa due to the edema [**22**].

## Paraclinical Diagnosis

- laboratory test results indicate leukocytosis.

- The abdominal X-ray on an empty stomach may show hydro air images, the segmental dilatation of the involved area, the presence of air at the level of the portal vein or the mesenteric vein, which represents a sign of severity, the imprinting in the involved colonic segments due to the intramucosal hemorrhages [**14**];

- The irrigography shows defects of nodular filling, edema of the mucosa, dehaustration of the colon, segmental stenoses, with supraadjacent dilatations [**13**];

- The colonoscopy does not have pathognomonic signs, but it has high sensitivity due to the involvement of the mucosa. Ischemia also induces secondary inflammatory lesions, so that the mucosa looks erythematous and edematous, with disseminated ulcerations. Black and bluish areas may appear which are secondary to the necrosis of the mucosa. Solitary colonic ulcer appears in 75% of the cases. After 48 hours from the onset, the mucosa becomes necrotic and it is hard to differentiate from Crohn’s disease or from pseudomembranous colitis. A characteristic of ischemic colitis is the non-involvement of the rectum, the segmental distribution of the lesions, the clear-cut delimitation of the involved lesions, the frequently favorable evolution towards healing [**23**];

- The findings of computed tomography are non-specific, they show the dilatation of the colon, with the segmental thickening of the colonic wall, the irregular narrowing of the lumen as a result of the edema, ascites, the presence of air in the portal vein or in the colonic wall;

- The ultrasound examination reveals a moderate thickening of the colonic wall, with the loss of the differentiation between its layers, and the absence of the arterial flow is a sign of severe prognosis [**12**];

- The mesenteric angiography is not a common investigation and it is performed only in the cases of acute ischemia;

- Computerized tomographic angiography detects the changes of the blood flow and of the secondary ischemic lesions.

**The anatomical pathologic examination** – is not pathognomonic, it shows the presence of macrophages loaded with hemosiderin [**10**].

**The differential diagnosis** is made between acute colitis caused by dysentery, the inflammatory diseases of the colon, diverticulitis [**24**].

Most frequently, the **prognosis** is favorable, in 50% of the cases it remits spontaneously within 48 hours, with full recovery within 14 days. There are severe cases with fever, shivering, bloody diarrhea which require an emergency laparatomy [**20**]. In rare cases the evolution is chronic and persistent [**19**].

## Complications

– may be local with perforations, bleeding, abscesses, peritonitis [**12**];

– systemic with heart failure, kidney failure, disseminated intravascular, coagulation, acidosis [**8**].

**Treatment** differs according to the clinical form. In general, it goes into remission spontaneously. The patients in a good state require feeding pause. The undernourished patients are administered parenteral alimentation, and those with severe forms are prescribed a feeding pause, hydroelectrolytic corrections, broad-spectrum antibiotics, transfusions in severe anemias, nasograstric tubes.

Analgesics, antispasmodic drugs and antidiarrheal medication are not used because they may lead to toxic megacolon. Corticotherapy predisposes to perforation of the colon and worsens ischemic lesions [**18**].

The emergency surgical treatment is required in acute pancolitis, toxic megacolon, massive hemorrhages, when a subtotal colectomy is performed. The surgical treatment may be temporized in the forms which evolve with segmental involvement, when the symptoms persist for more than 3 weeks, or in the cases of apparent healing with sepsis, when a segmental colectomy is performed, with a colostomy with the secondary repair of the continuity of the colon due to the increased risk of dehiscence of the primary anastomosis. In the case of colonic strictures, a segmental resection or an endoscopic dilation is performed [**27**].

## Conclusions

It is a relatively recent entity, well individualized from the clinical and therapeutical points of view, as it is the most frequent symptomatic ischemia, with an excellent prognosis in most of cases, whereas the surgical treatment is reserved only to the severe cases with complications.

## References

[1] Libby P (2002). Inflammation in atherosclerosis. Nature.

[2] van Leuven SI, Franssen R, Kastelein JJ, Levi M, Stroes ES, Tak PP (2008). Systemic inflammation as a risk factor for atherothrombosis. Rheumatology.

[3] López-Pedrera C, Pérez-Sánchez C, Ramos-Casals M, Santos-Gonzalez M, Rodriguez-Ariza A, José Cuadrado M (2012). Cardiovascular risk in systemic autoimmune diseases. epigenetic mechanisms of immune regulatory functions. Clin Dev Immunol.

[4] Zöller B, Li X, Sundquist J, Sundquist K (2011). Age- and gender-specific familial risks for venous thromboembolism: a nationwide epidemiological study based on hospitalizations in Sweden. Circulation.

[5] Rosen M, Hakulinen T (2005). Use of disease registers. Handbook of epidemiology.

[7] Rothman KJ, Greenland S (1998). Modern Epidemiology.

[8] Cheng YC, Wu CC, Lee CC, Lee TY, Hsiao KC (2012). Rare complication following screening colonoscopy: Ischemic colitis. Dig Endosc.

[9] Do BH, Jeon SW, Kim SG (2006). An analysis based on hospital stay in ischemic colitis. Korean J Gastrointest Endosc.

[10] Cha SB, Park SH, Cho SH (1996). Analysis of 15 cases of ischemic colitis induced by increased abdominal pressure. Korean J Gastrointest Endosc.

[11] Jin NC, Kim YJ, Song YA (2008). Clinical features of ischemic colitis: related to age, constipation, irritable bowel syndrome. Chonnam Med J.

[12] Jung SH, Lee KM, Ji JS (2008). Clinical features and prognostic factors in ischemic colitis. Korean J Gastrointest Endosc.

[13] MacDonald PH (2002). Ischaemic colitis. Best Pract Res Clin Gastroenterol.

[14] Scharff JR, Longo WE, Vartanian SM, Jacobs DL, Bahadursingh AN, Kaminski DL (2003). Ischemic colitis: spectrum of disease and outcome. Surgery.

[15] Lee TC, Wang HP, Chiu HM (2010). Male gender and renal dysfunction are predictors of adverse outcome in nonpostoperative ischemic colitis patients. J Clin Gastroenterol.

[16] Brandt LJ (2005). Bloody diarrhea in an elderly patient. Gastroenterology.

[17] Bower TC (1993). Ischemic colitis. Surg Clin North Am.

[18] Pla Martí V, Alós Company R, Ruiz Carmona MD, Solana Bueno A, Roig Vila JV (2001). Experience and results on the surgical and medical treatment of ischaemic colitis. Rev Esp Enferm Dig.

[19] Ahn SE, Lee HL, Cho SC (2009). Is end stage renal disease a poor prognosis factor of ischemic colitis?. Korean J Gastroenterol.

[20] Añón R, Boscá MM, Sanchiz V (2006). Factors predicting poor prognosis in ischemic colitis. World J Gastroenterol.

[21] Brandt LJ, Feuerstadt P, Blaszka MC (2010). Anatomic patterns, patient characteristics, and clinical outcomes in ischemic colitis: a study of 313 cases supported by histology. Am J Gastroenterol.

[22] Seok SJ, Kim KJ, Son BS (2009). Clinical features of ischemic colitis in patients with chronic kidney disease. Korean J Nephrol.

[23] Ryu KH, Shim KN, Jung SA (2006). Clinical features of ischemic colitis: a comparison with colonoscopic findings. Korean J Gastrointest Endosc.

[24] Brandt LJ, Boley SJ (2000). AGA technical review on intestinal ischemia. American Gastrointestinal Association. Gastroenterology.

[25] Taourel P, Aufort S, Merigeaud S, Doyon FC, Hoquet MD, Delabrousse E (2008). Imaging of ischemic colitis. Radiol Clin North Am.

[26] Paterno F, McGillicuddy EA, Schuster KM, Longo WE (2010). Ischemic colitis: risk factors for eventual surgery. Am J Surg.

[27] Barouk J, Gournay J, Bernard P, Masliah C, Le Neel JC, Galmiche JP (1999). Ischemic colitic in the elderly: predictive factors of gangrenous outcome. Gastroenterol Clin Biol.

